# Comparative outcomes of stereotactic body radiotherapy versus radiofrequency ablation in hepatocellular carcinoma within Milan criteria: a systematic review and meta-analysis

**DOI:** 10.3389/fonc.2025.1644001

**Published:** 2025-09-02

**Authors:** Jianxiong Jing, Furui Zhong, Hao Zhang, Guosong Luo, Zongzhi Du

**Affiliations:** ^1^ Department of Hepatobiliary and Pancreatic Surgery, Nanbu People’s Hospital, Nanchong, Sichuan, China; ^2^ Department of General Surgery, Zigong Fourth People’s Hospital, Zigong, Sichuan, China; ^3^ Department of Hepatobiliary and Pancreatic Surgery, Meishan People’s Hospital, Meishan, Sichuan, China; ^4^ Department of Center For Hepatobiliary-Pancreatic-Splenic Diseases, Zigong Fourth People’s Hospital, Zigong, Sichuan, China

**Keywords:** hepatocellular carcinoma, Milan criteria, stereotactic body radiation therapy, radiofrequency ablation, meta-analysis

## Abstract

**Objective:**

To systematically compare the clinical efficacy and adverse events between stereotactic body radiation therapy (SBRT) and radiofrequency ablation (RFA) in treating hepatocellular carcinoma (HCC) within the Milan criteria through a meta-analysis.

**Methods:**

A comprehensive literature search was conducted in PubMed, Embase, Cochrane Library, Scopus, and Web of Science from database inception to May 1, 2025, for studies comparing SBRT and RFA in HCC patients meeting the Milan criteria. Data were analyzed using RevMan 5.4 software for meta-analysis.

**Results:**

Ten studies (9 retrospective and 1 randomized controlled trial) involving 1505 patients were included. Pooled hazard ratios (HRs) for overall survival (OS: HR = 0.98, 95% CI = 0.72–1.32, P = 0.87) and progression-free survival (PFS: HR = 0.84, 95% CI = 0.67–1.06, P = 0.14) demonstrated no significant differences between SBRT and RFA. Subgroup analyses based on tumor diameter, tumor origin type, and study design revealed no significant differences in pooled HRs for OS or PFS. The incidence of adverse events showed no statistical difference between SBRT and RFA (RR = 0.73, 95% CI = 0.53–1.01, P = 0.05).

**Conclusion:**

SBRT and RFA exhibit comparable efficacy and safety profiles in managing HCC within the Milan criteria.

## Introduction

Hepatocellular carcinoma (HCC), a primary liver malignancy ranking sixth in global incidence and third in cancer-related mortality, imposes a substantial disease burden (1). Its pathogenesis predominantly arises in the context of chronic liver diseases, with major etiological factors including chronic hepatitis B/C virus infections, alcoholic cirrhosis, and non-alcoholic fatty liver disease (2). Surgical resection and liver transplantation remain the gold-standard curative therapies for early-stage HCC (3, 4), particularly in patients meeting Milan criteria (5). These interventions achieve 5-year survival rates ranging from 60% to 80% (6), earning them first-line recommendations in guidelines from the European Association for the Study of the Liver (EASL,2024) (4) and the American Association for the Study of Liver Diseases (AASLD, 2023) (7).

However, 30–40% of patients are precluded from these radical surgical options due to clinical constraints (8) such as inadequate hepatic functional reserve (9), complex tumor anatomy such as proximity to major vasculature or hepatic hilum (10), tumor diameter exceeding eligibility thresholds (11), or donor organ shortages (12). For intermediate-stage patients ineligible for surgery, the BCLC guidelines recommend transarterial chemoembolization (TACE) as standard of care (13), while radioembolization using yttrium-90 microspheres is recommended for preserving liver function in advanced cases (14). Radiofrequency ablation (RFA) serves as a principal locoregional therapy for early-stage tumors not amenable to surgery, achieving local control rates of 70–90% (15). Nevertheless, its efficacy exhibits significant dependence on cirrhosis severity, tumor size (particularly lesions >3 cm), and anatomical location (4). Technical limitations include incomplete ablation and procedure-related complications such as needle tract seeding metastasis, intrahepatic hemorrhage, and thermal injury to adjacent organs (16, 17).

Notably, stereotactic body radiation therapy (SBRT) as an emerging local treatment modality in recent years, has demonstrated the ability to effectively control tumor growth while minimizing damage to surrounding normal tissues (18), owing to its submillimeter positioning accuracy and steep dose gradient characteristics (19). Recent clinical observations have highlighted its potential for HCC, particularly for lesions abutting ablation-sensitive organs such as the gastrointestinal tract and diaphragm (20). Research conducted by Fu et al. (21) and Maher et al. (22) have both demonstrated that for patients meeting the Milan criteria, the overall survival (OS) and progression-free survival (PFS) of SBRT are comparable to those of RFA. Conversely, a propensity score matching study conducted by Berger et al. (23) revealed median survival times of 22 months for the SBRT group and 32 months for the RFA group, with the OS in the SBRT cohort being significantly lower than that in the RFA cohort.

Existing studies comparing SBRT and RFA are largely defined by single-center retrospective designs, limited sample sizes, and heterogeneous inclusion criteria, resulting in a scarcity of high-quality evidence for survival outcomes and treatment-related adverse events in HCC patients meeting Milan criteria. This meta-analysis specifically focuses on patients within Milan criteria because both RFA and SBRT are validated treatment options for this subgroup in EASL/AASLD guidelines (4, 7). By systematically evaluating OS, PFS, and adverse event profiles, this study aims to provide high-quality evidence to guide precise selection of curative local treatment protocols in clinical practice.

## Methods

### Search strategy

This study adhered to the Preferred Reporting Items for Systematic Reviews and Meta-Analyses (PRISMA) 2020 guidelines (24), employing a systematic literature search strategy. Computerized searches were conducted in databases including PubMed, Embase, Cochrane Library, Scopus, and Web of Science, spanning from the inception of each database to May 1, 2025. A comprehensive search strategy combining Medical Subject Headings (MeSH) and free-text keywords was utilized to enhance retrieval precision. Boolean operators (AND/OR) were used for logical term combination, with adaptations made to align with the subject indexing rules of each database. For example, the PubMed search strategy employed the following syntax: ((“Hepatocellular Carcinoma”[Mesh] OR hepatocellular carcinoma OR HCC) AND (“Stereotactic Body Radiation Therapy”[Mesh] OR stereotactic body radiotherapy OR SBRT) AND (“Radiofrequency Ablation”[Mesh] OR radiofrequency ablation OR RFA)). To minimize the risk of potentially eligible studies being overlooked, a manual search of the reference lists of included studies was conducted as a supplementary search strategy.

### Inclusion and exclusion criteria

Inclusion criteria for studies:

1. Study Population: Adult patients with pathologically or radiologically confirmed HCC meeting the Milan criteria (single tumor ≤5 cm in diameter, or ≤3 tumors with the largest diameter ≤3 cm, without vascular invasion or extrahepatic metastasis).
2. Interventions: Experimental group receiving SBRT as the primary treatment modality, with the control group undergoing RFA.
3. Study Design: Randomized controlled trials (RCTs) or prospective/retrospective cohort studies that directly compare the outcomes of SBRT and RFA.
4. Outcome Measures: At least one of the following prognostic data reported: OS, PFS, or incidence of adverse events.
5. Data Completeness: Extractable survival analysis data (e.g., hazard ratios [HR], 95% confidence intervals [CI], or Kaplan-Meier curves) provided.

Exclusion Criteria for Studies:

1. Non-comparative studies, case reports, conference abstracts, review articles, or animal studies.
2. Studies involving mixed HCC stages or treatment modalities that preclude separate extraction of SBRT and RFA data.
3. Duplicate publications or studies with overlapping data (only the study with the largest sample size or longest follow-up duration was retained).
4. Non-English publications or studies with available only as abstracts without full texts.
5. Studies with a sample size <20 cases or follow-up duration <12 months.

### Data extraction and research quality evaluation

Literature screening and data extraction were independently performed by two investigators (J.X.J and F.R.Z) trained in systematic review methodology. Literature was managed by use Zotero 6.0 software. Discrepancies were resolved through cross-checking and discussion, with a third investigator (G.S.L) invited for arbitration when necessary. Data extraction included basic characteristics such as first author, publication year, study type, country, number of samples, gender, age, tumor diameter and Child-Pugh grading; intervention parameters including SBRT total dose and fractionation scheme; and outcome data comprising OS, PFS, and incidence of treatment-related adverse events. Retrospective studies using propensity score matching (PSM) and RCTs were both considered to have a high level of research quality evidence.

The Risk of Bias In Non-randomized Studies of Interventions (ROBINS-I) tool (25) was employed to evaluate the risk of bias in the included non-randomized controlled studies. Two investigators independently conducted these assessments, focusing on seven specific domains: confounding, selection of participants, exposure assessment, misclassification during follow-up, missing data, measurement of the outcome, and selective reporting of the results. The risk of bias was categorized as “low risk,” “moderate risk,” “high risk,” or “uncertain risk” based on the strength of the evidence. For RCTs, the Cochrane-recommended Risk of Bias 2 (RoB 2) tool (26) was utilized, addressing the following domains: the randomization process, deviations from intended interventions, missing data; outcome measurement, and selective reporting. Ratings were classified as “low risk,” “some concerns,” or “high risk” according to evidence strength. Inter-rater agreement was quantified using the weighted Cohen’s kappa coefficient, and discrepancies were resolved through consultation with a third investigator to reach consensus.

### Statistical analysis

Meta-analysis was performed using RevMan 5.4 software. For dichotomous outcomes, risk ratios (RR) with 95% confidence intervals (CI) were employed as effect measures. Survival endpoints (OS and PFS) were analyzed using hazard ratios (HR). In studies where HRs were not explicitly reported, values were derived from Kaplan-Meier survival curves through digitization and calibration of coordinate points using Engauge Digitizer 4.1 software, following the methodology proposed by Tierney et al. (27). Heterogeneity was quantified using the χ² test (Cochran’s Q statistic) and I² statistic. A fixed-effect model (Mantel-Haenszel method) was applied when heterogeneity was low (P > 0.1 for χ² test and I² < 50%). For high heterogeneity (P ≤ 0.1 or I² ≥ 50%), subgroup analyses and sensitivity analyses were conducted to explore heterogeneity sources. If unresolved, a random-effects model (DerSimonian-Laird method) was adopted. Subgroup investigations stratified studies by tumor diameter (≤3 cm *vs*. 3–5 cm), tumor origin types (primary *vs*. recurrent), and study design (RCT/PSM study *vs*. retrospective study). Sensitivity analyses were conducted using a leave-one-out approach to determine if the pooled effect sizes were significantly affected by individual studies. Publication bias was assessed through a visual examination of the funnel plot symmetry. All statistical tests were two-sided, with a significance level set at α=0.05.

## Result

### Literature retrieval results

**Figure 1:**
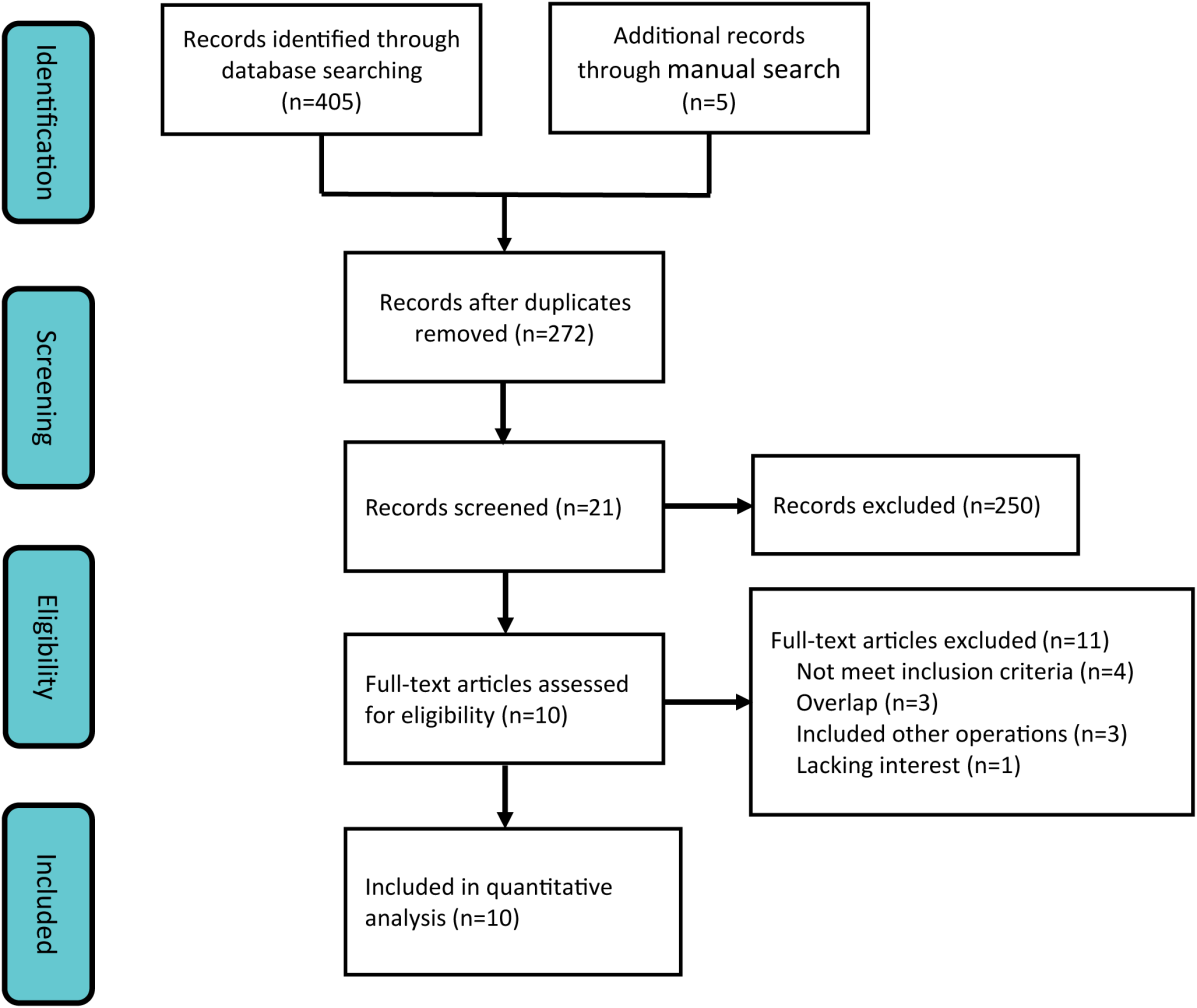
Flowchart of selection of studies included in the meta-analysis.

Employing a systematic search strategy, we initially identified 410 potentially pertinent studies. Through a stepwise screening process, which involved the exclusion of duplicate publications, review articles, case reports, single-arm studies, and animal experiments, 21 studies satisfied the preliminary inclusion criteria. Subsequent to a comprehensive full-text review, 11 additional studies were excluded due to non-compliance with the predefined outcome indicators. Consequently, 10 clinical studies (21, 22, 28–35) were incorporated into the meta-analysis (**Figure 1**), consisting of 1 RCT and 9 retrospective studies.

### Characteristics and methodological quality assessment of included studies

The studies incorporated in this meta-analysis were published between 2015 and 2024, encompassing five studies from China (21, 28, 30, 31, 34), three from Japan (29, 32, 33), one from South Korea (35), and one from Australia (29). Collectively, these studies included a total of 1505 patients diagnosed with hepatocellular carcinoma who met the Milan criteria. Of these patients, 667 were assigned to the SBRT group, while 838 were assigned to the RFA group. Baseline characteristic analyses revealed no statistically significant differences between SBRT and RFA cohorts across demographic parameters including gender distribution, patient age, and pre-treatment liver function status. Notably, in two comparative studies (28, 29), the SBRT groups exhibited significantly larger tumor diameters compared to their RFA counterparts, suggesting potential treatment selection bias (**Table 1**). With regard to SBRT protocols, the administered biologically effective doses varied between participating institutions, ranging from 27.5 to 112.5 Gy, with fractionation schedules typically delivered over 3 to 5 consecutive treatment sessions.

**Table 1:** The characteristics of the studies included in the meta-analysis.

Study	Country	Study type	Number of samples (n)		Gender (male/female)		Age (years)		Tumor diameter (cm)		Child-Pugh Grading (A/B)		SBRT scheme
			SBRT	RFA	SBRT	RFA	SBRT	RFA	SBRT	RFA	SBRT	RFA	
Shiozawa 2015 (29)	Japan	n-RCT	35	38	24/11	27/11	75.1 ± 8.1	68.7 ± 10.5	2.88 ± 1.2	1.7 ± 0.6	28/7	31/7	Total dose: 60 Gy; 3 to 5 fractions.
Hara 2019 (32)	Japan	PSM	106	106	71/35	76/30	74 ± 11.2	75 ± 10.2	1.8 ± 0.5	1.7 ± 0.5	105/1	104/2	Total dose: 35 or 40 Gy; 5 fractions.
Ueno 2021 (33)	Japan	PSM	31	62	24/7	43/19	75.2 ± 9.1	75.0 ± 7.5	1.5 ± 0.6	1.5 ± 0.5	NA	NA	Total dose: 40 Gy; 5 fractions.
Ji 2022 (28)	China	n-RCT	22	38	15/7	31/7	66.5 ± 13	61.5 ± 8.5	4.35 ± 1	1.8 ± 0.7	21/1	35/3	Total dose: 27.5–50 Gy; 5 fractions.
Shin 2022 (35)	South Korea	n-RCT	72	134	46/26	102/32	62.7 ± 10.8	60.3 ± 10.4	1.7 ± 0.6	1.6 ± 0.5	NA	NA	Total dose: 40–60 Gy; 3 to 5 fractions.
Maher 2024 (22)	Australia	n-RCT	36	51	27/9	41/10	68.1 ± 7.6	62.6 ± 8.8	2.1 ± 0.4	1.8 ± 0.3	NA	NA	Total dose: 48-112.5 Gy; 3 to 5 fractions.
Yang 2024 (34)	China	n-RCT	122	166	106/16	146/20	56.0 ± 9.6	55.0 ± 11.1	2.3 ± 1	2.1 ± 0.8	120/2	165/1	Total dose: 36–54 Gy; 3 fractions.
Fu 2025 (21)	China	PSM	118	118	103/15	99/19	55.8 ± 11.2	54.4 ± 12.7	2.4 ± 0.9	2.3 ± 0.8	118/0	118/0	Total dose: 30–54 Gy; 3 fractions.
Ma 2025 (30)	China	PSM	42	42	36/6	36/6	NA	NA	NA	NA	41/1	39/3	Total dose: 37.5 or 49 Gy; 3 to 5 fractions.
Xi 2025 (31)	China	RCT	83	83	74/9	78/5	56.0 ± 10.4	58.0 ± 9.6	1.7 ± 0.7	1.6 ± 0.7	83/0	83/0	Total dose: 36–54 Gy; 3 fractions.

SBRT, stereotactic body radiation therapy; RFA, radiofrequency ablation; NA, not applicable; RCT, randomized controlled trials; PSM, propensity score matching.

The methodological quality of the single randomized controlled trial (RCT) was assessed using the RoB 2 tool. The evaluation revealed “some concerns” in the domain of deviations from intended interventions, attributed to incomplete reporting of blinding implementation details, while all other domains were rated as “low risk.” Consequently, the overall assessment classified this RCT as having “some concerns” regarding methodological quality (**Supplementary Figure 1**). For the nine non-RCTs, the risk of bias was evaluated using the ROBINS-I tool. Specifically, three studies were rated as having a “moderate risk” for confounding bias control, and three for outcome measurement bias. In the domain of selection bias, one study was deemed to have a “moderate risk” due to unclear inclusion criteria. Regarding bias due to missing data, two studies failed to report follow-up loss. Importantly, no studies were rated as “high risk” in the overall rating. The consistency between the two evaluators was relatively high (Cohen’s kappa=0.759), and the main differences focused on the determination of bias in exposure assessment and measurement of the outcome (**Supplementary Table 1**). Finally, a consensus was reached after third-party arbitration.

### Overall survival

**Figure 2:**
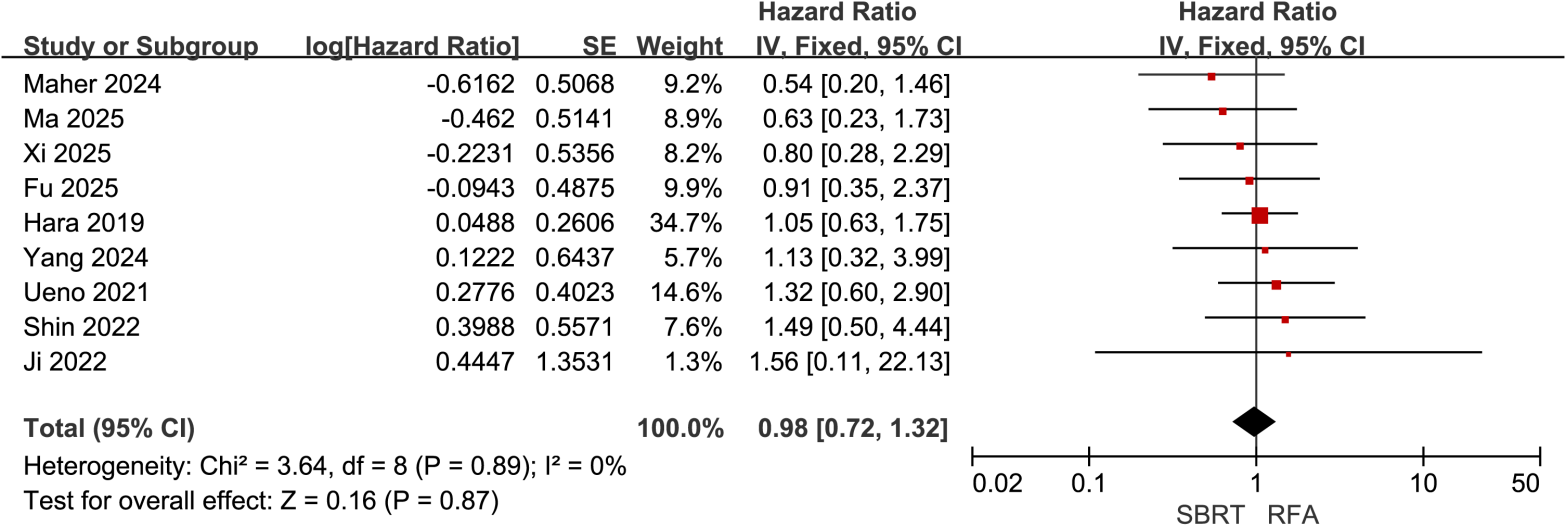
Forest plot of hazard ratios (HR) for overall survival (OS) comparing the Stereotactic Body Radiotherapy (SBRT) and Radiofrequency Ablation (RFA) groups in patients with Hepatocellular Carcinoma (HCC) within Milan criteria.

Nine of the included studies (21, 22, 28, 30–35) compared OS between SBRT and RFA for HCC patients meeting the Milan criteria, with 632 patients in the SBRT group and 800 in the RFA group. Heterogeneity testing indicated low inter-study heterogeneity (I² = 0%). A fixed-effect model was selected for meta-analysis, which demonstrated no significant difference in OS between the two groups (HR = 0.98, 95% CI = 0.72–1.32, P = 0.87) (**Figure 2**).

### Disease free survival

**Figure 3:**
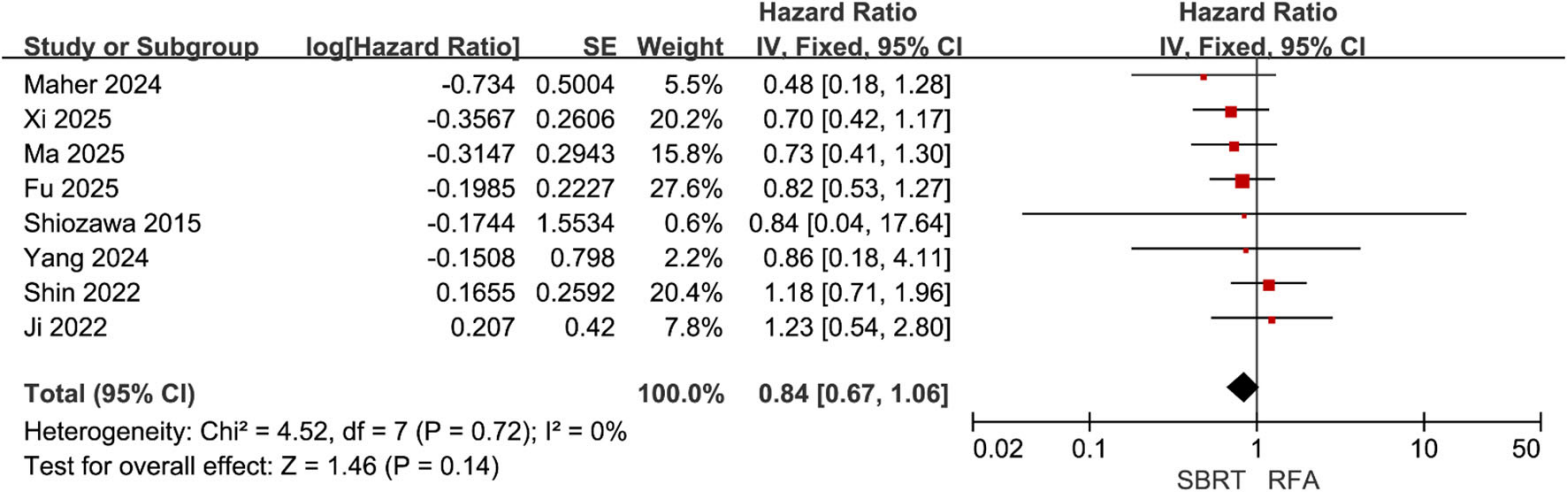
Forest plot of hazard ratios (HR) for progression-free survival (PFS) comparing the Stereotactic Body Radiotherapy (SBRT) and Radiofrequency Ablation (RFA) groups in patients with Hepatocellular Carcinoma (HCC) within Milan criteria.

Eight of the included studies (21, 22, 28–31, 34, 35) compared PFS between SBRT and RFA for HCC patients meeting the Milan criteria, with 530 patients in the SBRT group and 670 in the RFA group. Heterogeneity testing revealed low inter-study heterogeneity (I² = 0%). A fixed-effect model was selected for meta-analysis, which demonstrated no significant difference in PFS between the two groups (HR = 0.84, 95% CI = 0.67–1.06, P = 0.14) (**Figure 3**).

### Subgroup analysis

The subgroup analysis results indicated that across various stratifications of tumor diameter (≤3 cm and 3–5 cm), tumor origin types (primary HCC, recurrent HCC, and not distinguished), and study design types (RCT/PSM study or retrospective study), there were no statistically significant differences in the HR for OS and PFS between the SBRT and RFA groups (all P values > 0.05) (**Table 2**).

**Table 2:** Subgroup meta-analysis comparing OS and PFS between SBRT and RFA.

Survival Situation	Subgroup	Number	HR	95%CI	P-value
OS	Tumor diameter				
	≤3cm	6	1.01	0.71-1.45	0.94
	3-5cm	2	0.55	0.03-12.13	0.71
	Tumor origin				
	Primary HCC	3	0.68	0.28-1.64	0.39
	Recurrent	2	0.71	0.34-1.46	0.35
	Not distinguished	4	1.09	0.75-1.58	0.66
	Study design				
	RCT/PSM	5	0.99	0.70-1.39	0.93
	Retrospective	4	0.94	0.51-1.75	0.86
PFS	Tumor diameter				
	≤3cm	6	0.89	0.69-1.15	0.37
	3-5cm	4	0.98	0.62-1.54	0.93
	Tumor origin				
	Primary HCC	5	1.02	0.72-1.46	0.9
	Recurrent	3	0.74	0.55-1.01	0.06
	Not distinguished	1	0.86	0.58-1.28	0.45
	Study design				
	RCT/PSM	3	0.76	0.57-1.01	0.06
	Retrospective	5	1.02	0.69-1.49	0.94

OS: overall survival; PFS: progression-free survival; HR: hazard ratios;

### Adverse event

**Figure 4:**
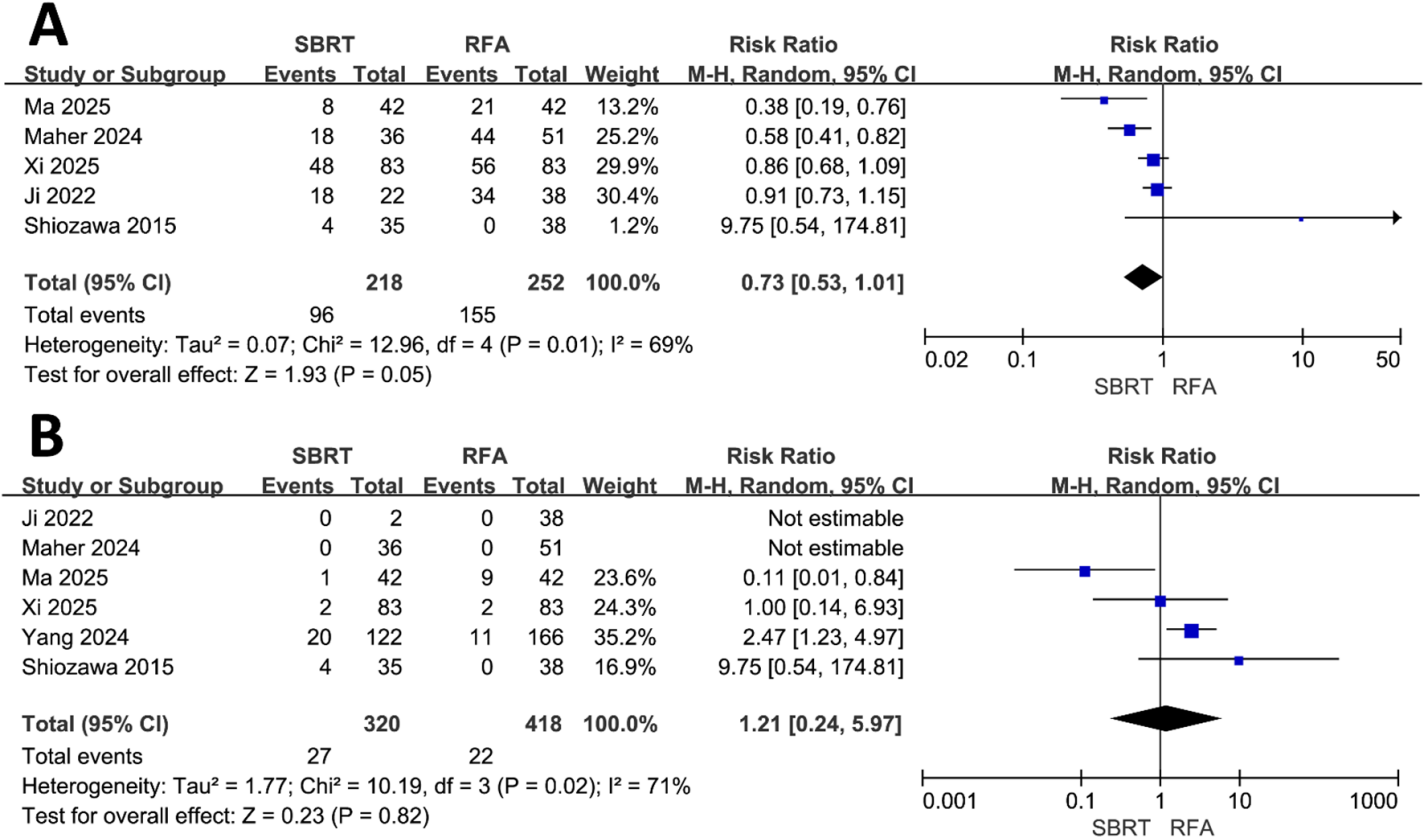
Forest plot of RR of any adverse events **(A)** and serious adverse events **(B)** comparing the SBRT and RFA groups in patients with HCC within Milan criteria.

Five studies (22, 28–31) documented adverse event outcomes, identifying 96 adverse events among 218 patients in the SBRT group and 155 adverse events among 252 patients in the RFA group. The meta-analysis indicated no statistically significant difference in the incidence of adverse events between the groups (RR = 0.73, 95% CI = 0.53–1.01, P = 0.05) (**Figure 4A**). Furthermore, six studies (22, 28–31, 34) reported serious adverse events (CTCAE grade ≥3), with 27 events occurring in 320 patients in the SBRT group and 22 events in 418 patients in the RFA group. The meta-analytic findings revealed no significant difference between the groups in the incidence of serious adverse events (RR = 1.48, 95% CI = 0.88–2.49, P = 0.14) (**Figure 4B**).

### Publication bias and sensitivity analysis

**Figure 5:**
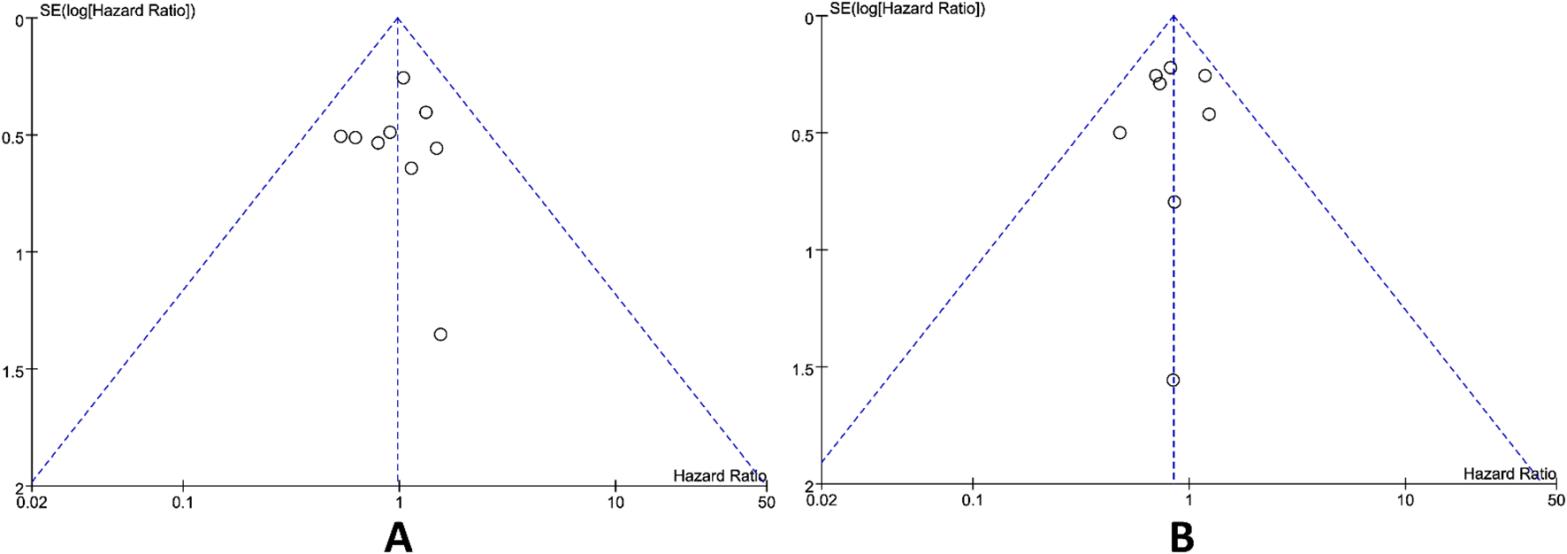
Funnel plots for assessing publication bias in primary outcomes of OS **(A)** and PFS **(B)**.

Publication bias was assessed utilizing the funnel plot method. Upon visual inspection, the funnel plots for the primary outcomes OS and PFS was appeared approximately symmetrical (**Figure 5**), suggesting that publication bias exerted a minimal influence on the results. Sensitivity analysis conducted by leave-one-out method demonstrated that excluding any single study did not significantly alter the pooled effect sizes, indicating the meta-analysis results were highly robust and unaffected by individual studies.

## Discussion

This meta-analysis systematically evaluated the therapeutic efficacy and safety of SBRT versus RFA in HCC patients meeting Milan criteria. The findings demonstrated comparable efficacy between the two modalities in OS (HR = 0.98, 95% CI = 0.72–1.32, P = 0.87) and PFS (HR = 0.84, 95% CI = 0.67–1.06, P = 0.14), with SBRT showing no statistically significant increase in treatment-related adverse events (RR = 0.73, 95% CI = 0.53–1.01, P = 0.05). These results provide critical clinical decision-making insights for non-surgical candidates with Milan criteria-compliant HCC, positioning SBRT as an alternative treatment option that is non-inferior to RFA. This conclusion significantly broadens the spectrum of local therapeutic options available for HCC management, particularly in scenarios where ablation may be contraindicated or technically challenging.

As an established curative-intent local therapy for HCC, RFA has definite therapeutic effects on HCC that meets the Milan criteria (36). However, its clinical application encounters significant challenges when tumors are located in subcapsular regions, adjacent to the diaphragmatic dome, or in proximity to major vascular structures (17). In such challenging anatomical contexts, RFA frequently encounters technical limitations including heat sink effects (37) and restricted maneuverability, resulting in incomplete ablation rates ranging from 7.2% to 34.7% (38). These suboptimal outcomes often necessitate salvage interventions such as transarterial chemoembolization or repeat ablation procedures (39). Historically, conventional external beam radiotherapy has been limited in patients with cirrhosis due to the risk of radiation-induced liver disease and inadequate dose conformity (40, 41). In contrast, SBRT addresses these technical challenges by utilizing four-dimensional computed tomography simulation with respiratory gating (32), which allows for precise control of liver motion within a 3 mm displacement (42). Advanced inverse planning intensity-modulated techniques facilitate steep dose gradients of 10% per millimeter (43), thereby restricting radiation exposure to ≤15 Gy in normal hepatic parenchyma within 2 cm of the target volume (44). This precise dosimetry significantly reduces the likelihood of radiation-induced liver injury while ensuring therapeutic doses are delivered to tumor targets (29). Consequently, these technological advancements render SBRT a viable alternative for patients with lesions contraindicated for radiofrequency ablation (RFA) or those facing elevated procedural risks (28, 30), particularly in anatomically challenging situations where thermal ablation is less effective.

RFA achieves tumor control primarily through direct thermal effects that induce coagulative necrosis (36), whereas SBRT relies on high-dose radiation to provoke tumor vascular damage and DNA double-strand breaks (18). Despite their divergent mechanisms of action, the equivalent survival outcomes observed in this study suggest that for patients meeting the Milan criteria, treatment selection should prioritize individualized considerations—such as tumor anatomical location, hepatic functional reserve, and procedural feasibility—rather than pursuing marginal survival differences (4). Subgroup analyses demonstrated internal consistency in the study findings. No significant heterogeneity in HRs was observed between tumor diameter stratifications (≤3 cm *vs*. 3–5 cm), indicating comparable tumor burden control efficacy between the two modalities. This contrasts with prior reports by Xi et al. (31), which suggested superior local control with SBRT for smaller tumors (≤3 cm). However, our analysis incorporated additional RCTs and PSM studies, thereby mitigating selection bias and providing a more objective reflection of real-world efficacy. Notably, consistent outcomes were observed in both primary and recurrent HCC subgroups, implying that prior treatment history may not influence the relative efficacy of these modalities. This finding holds significant clinical implications for patients with post-transplant recurrence or disease progression after prior locoregional therapies.

In this study, no significant difference in treatment-related adverse event rates was observed between the SBRT and RFA groups, which contrasts with previous reports suggesting a potential link between SBRT and increased risks of radiation-induced liver disease (45). Notably, however, the comparative analysis of adverse events revealed borderline statistical significance (RR = 0.73, 95% CI = 0.53-1.01, P = 0.05), a finding that warrants careful consideration. This borderline result hints at a subtle trend toward potentially increased toxicity with SBRT, though the narrow confidence interval (spanning close to 1.0) indicates that the absolute difference in clinical risk between the two modalities remains limited and may not reach meaningful clinical relevance. Several factors may contribute to this nuanced observation. First, contemporary advancements in radiotherapy techniques have likely played a pivotal role in mitigating excessive toxicity risks. Innovations such as image-guided radiotherapy (46), respiratory gating (47), and dose sculpting algorithms (19) have significantly improved the precision of radiation delivery, thereby minimizing inadvertent exposure to healthy hepatic parenchyma. Second, the included studies in our analysis consistently applied stringent dosimetric constraints, which are known to reduce the likelihood of radiation-induced liver injury. These technical refinements and standardized safety protocols may explain why the observed trend toward increased toxicity with SBRT remains borderline and clinically modest, rather than reaching the more pronounced risk levels suggested in earlier literature.

These findings collectively highlight the evolving role of technological advancements in reducing toxicity risks associated with SBRT (48), thereby challenging historical concerns regarding its safety compared to RFA in specific patient populations.

Several limitations warrant attention. First, difficulties in accessing raw data hindered the performance of subgroup analyses for critical variables, such as the number of liver tumors, cirrhosis status, RFA margin width, and SBRT biologically effective dose, as well as specific data on subsequent targeted or immunotherapy regimens. This limitation may impede the ability to identify context-specific survival benefits associated with each technique, particularly within high-risk subgroups. Secondly, most studies lack detailed data on tumor location, a factor that may significantly influence local recurrence rates and the risk of complications. Lastly, our meta-analysis did not address cost-effectiveness analyses and quality-of-life assessments, both of which are essential for a comprehensive and objective comparison of the clinical value of the two techniques.

## Conclusion

This study addresses the lack of direct comparative evidence between stereotactic body radiotherapy (SBRT) and radiofrequency ablation (RFA) in treating hepatocellular carcinoma (HCC) under the Milan criteria. Using a systematic meta-analysis, it evaluated the clinical efficacy and safety of both treatments. The results indicate that SBRT and RFA are statistically equivalent in key efficacy measures like overall and disease-free survival rates, as well as in safety indicators such as complication rates. The study suggests SBRT as a viable alternative to RFA, particularly for patients with tumors in challenging locations, and highlights its potential in HCC treatment. This supports SBRT as a reliable option for personalized HCC diagnosis and treatment strategies.

## Data Availability

The original contributions presented in the study are included in the article/**Supplementary Material**. Further inquiries can be directed to the corresponding authors.

## Supplementary material

Supplementary Table 1 Risk of bias for the included the nine non-RCTs, based on the ROBINS-I tool. ROBINS-I: risk of bias in non-randomized studies of interventions; NA=not applicable

Supplementary Figure 1 Risk of bias for the included the one RCT, based on the RoB 2 tool.
