# Supplementary material for: Comparative outcomes of stereotactic body radiotherapy versus radiofrequency ablation in hepatocellular carcinoma within Milan criteria: a systematic review and meta-analysis
Source: Front Oncol. 2025 Sep 2;15:1644001. doi: 10.3389/fonc.2025.1644001 (PMC12436108; doi:10.3389/fonc.2025.1644001)

| Table S1 Risk of bias for the included the nine non-RCTs, based on the ROBINS-I tool | | | | | | | | | |
| --- | --- | --- | --- | --- | --- | --- | --- | --- | --- |
| Author | Year | Type of bias | | | | | | | Overall rating |
|  |  | Confounding | Selection of participants | Exposure assessment | Misclassification during follow-up | Missing data | Measurement of the outcome | Selective reporting of the results |  |
| Shiozawa | 2015 | Moderate | Moderate | Low | Moderate | Low | Moderate | Low | Moderate |
| Hara | 2019 | Low | Low | Low | Low | Low | Low | Low | Low |
| Ueno | 2021 | Moderate | Low | Low | Moderate | Low | Low | Low | Moderate |
| Ji | 2022 | Low | Low | Low | Low | Low | Low | Low | Low |
| Shin | 2022 | Low | Low | Low | Low | Low | Moderate | Low | Moderate |
| Maher | 2024 | Moderate | Low | Low | Low | Moderate | Low | Low | Moderate |
| Yang | 2024 | Low | Low | Low | Low | Moderate | Moderate | Low | Moderate |
| Fu | 2025 | Low | Low | Low | Low | Low | Low | Low | Low |
| Ma | 2025 | Low | Low | Low | Low | Low | Low | Low | Low |
| Kappa | NA | 1 | 1 | 0.76 | 1 | 1 | 0.76 | 1 | 0.76 |

ROBINS-I: risk of bias in non-randomized studies of interventions; NA=not applicable

Supplementary Figure 1 Risk of bias for the included the one RCT, based on the RoB 2 tool


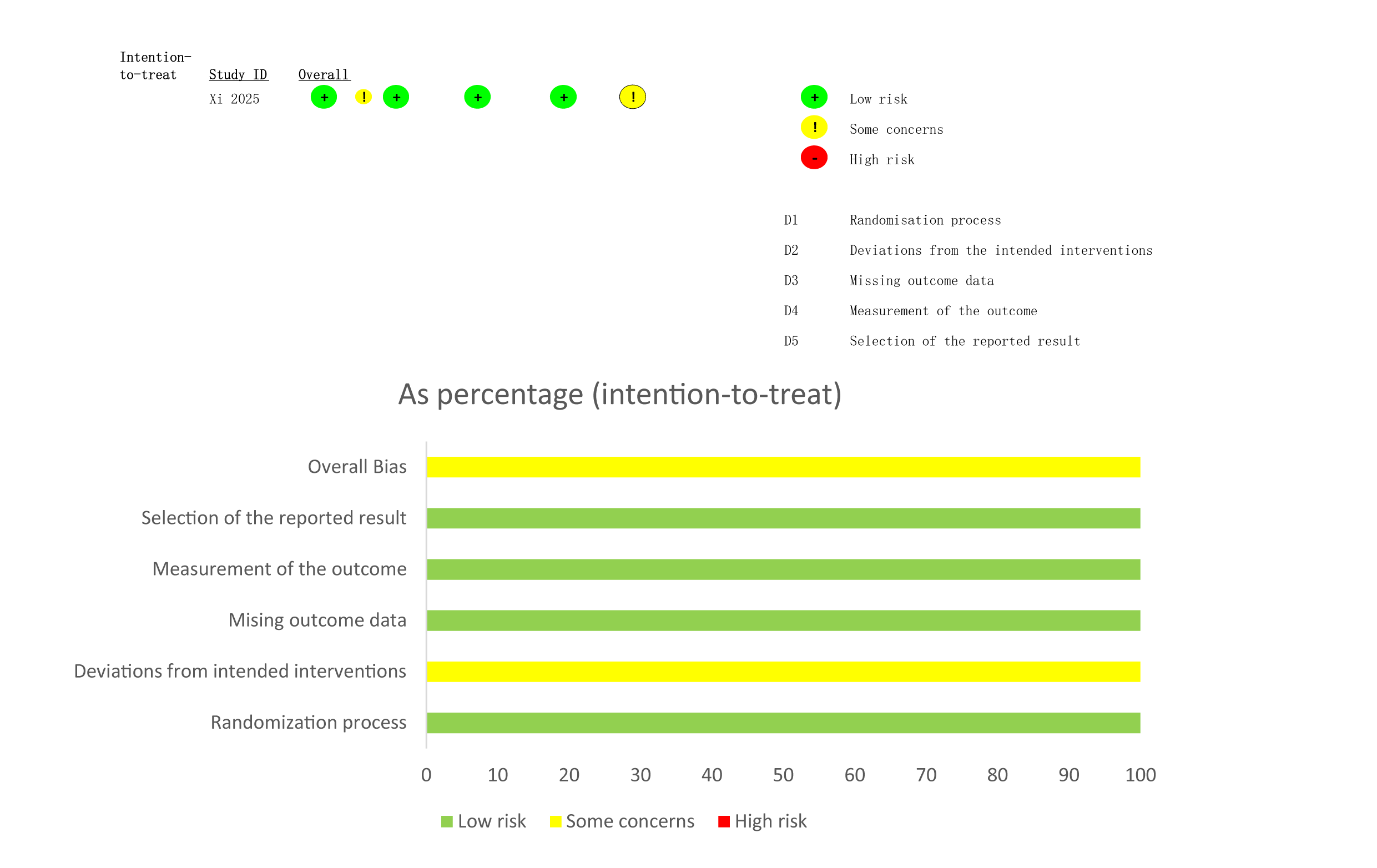

Supplement: Supplementary Table 1 — Risk of bias for the included the nine non-RCTs, based on the ROBINS-I tool. ROBINS-I: risk of bias in non-randomized studies of interventions; NA=not applicable [file Supplementaryfile1.docx]
